# Effect of Redox Potential on Diiron-Mediated Disproportionation of Hydrogen Peroxide

**DOI:** 10.3390/molecules28072905

**Published:** 2023-03-23

**Authors:** Patrik Török, Dóra Lakk-Bogáth, József Kaizer

**Affiliations:** Research Group of Bioorganic and Biocoordination Chemistry, University of Pannonia, H-8201 Veszprém, Hungary; patriktrk6@gmail.com (P.T.); lakkd@almos.uni-pannon.hu (D.L.-B.)

**Keywords:** nonheme models, *μ*-1,2-peroxo-diiron(III) intermediates, catalase-like activity, kinetics

## Abstract

Heme and nonheme dimanganese catalases are widely distributed in living organisms to participate in antioxidant defenses that protect biological systems from oxidative stress. The key step in these processes is the disproportionation of H_2_O_2_ to O_2_ and water, which can be interpreted via two different mechanisms, namely via the formation of high-valent oxoiron(IV) and peroxodimanganese(III) or diiron(III) intermediates. In order to better understand the mechanism of this important process, we have chosen such synthetic model compounds that can be used to map the nature of the catalytically active species and the factors influencing their activities. Our previously reported *μ*-1,2-peroxo-diiron(III)-containing biomimics are good candidates, as both proposed reactive intermediates (Fe^IV^O and Fe^III^_2_(*μ*-O_2_)) can be derived from them. Based on this, we have investigated and compared five heterobidentate-ligand-containing model systems including the previously reported and fully characterized [Fe^II^(L^1−4^)_3_]^2+^ (L^1^ = 2-(2′-pyridyl)-1*H*-benzimidazole, L^2^ = 2-(2′-pyridyl)-*N*-methyl-benzimidazole, L^3^ = 2-(4-thiazolyl)-1*H*-benzimidazole and L^4^ = 2-(4′-methyl-2′-pyridyl)-1*H*-benzimidazole) and the novel [Fe^II^(L^5^)_3_]^2+^ (L^5^ = 2-(1*H*-1,2,4-triazol-3-yl)-pyridine) precursor complexes with their spectroscopically characterized *μ*-1,2-peroxo-diiron(III) intermediates. Based on the reaction kinetic measurements and previous computational studies, it can be said that the disproportionation reaction of H_2_O_2_ can be interpreted through the formation of an electrophilic oxoiron(IV) intermediate that can be derived from the homolysis of the O–O bond of the forming *μ*-1,2-peroxo-diiron(III) complexes. We also found that the disproportionation rate of the H_2_O_2_ shows a linear correlation with the Fe^III/^Fe^II^ redox potential (in the range of 804 mV-1039 mV vs. SCE) of the catalysts controlled by the modification of the ligand environment. Furthermore, it is important to note that the two most active catalysts with L^3^ and L^5^ ligands have a high-spin electronic configuration.

## 1. Introduction

Iron and manganese are the most important candidates for both oxygen activation and environmental sustainability. Both metal ions have a variety of oxidation states that can be easily tuned in terms of Lewis acidity and hardness/softness, which can lead to fine-tuning of redox systems. Catalases (including hydrogen peroxide oxidoreductase) are important antioxidants necessary for life in oxygen-metabolizing cells to regulate H_2_O_2_ concentration by accelerating the disproportionation of a toxic oxygen metabolite, hydrogen peroxide, forming dioxygen and water [1,2,3,4,5]. H_2_O_2_ as reactive oxygen species (ROS) can easily be formed enzymatically by oxidases and non-enzymatically as a byproduct of respiration or autoxidation of cellular components. Most physio-pathological situations such as neurodegenerative diseases (Parkinson’s disease and Alzheimer’s disease), chronic inflammatory diseases, chronic obstructive pulmonary diseases (COPD), chronic kidney diseases (CKD), metabolic diseases (diabetes) or cancers can be associated with oxidative stress resulting from H_2_O_2_ [6,7,8,9,10,11,12,13,14]. Hydrogen peroxide exerts its toxic effects in several ways, including the activation of transition metal ions to Fenton chemistry, generating hydroxyl radicals that can damage of various biomolecules such as lipids, proteins or DNA and even cellular death [15,16,17,18].

Catalase enzymes are found in both aerobic prokaryotic and eukaryotic cells of aerobic organisms. Iron is an inorganic cofactor essential for many organisms. Bacteria adapt to the environment, which limits the availability of iron and the use of other metals such as manganese ions for their essential functions. Two families of catalases are known, one having a heme cofactor and the other a structurally distinct family containing nonheme manganese such as *L. plantarum* and *T. thermophilus* [1].

Heme-containing (type I) catalases are mostly found in aerobic organisms and have been characterized both structurally and biochemically. These enzymes bind an iron-porphyrin (FeP) redox cofactor in their active site, which undergoes a two-electron change in its oxidation state during the transformation from Fe(III)P to an Fe(IV)OP• oxo-ferryl porphyrin π-radical complex (Compound I) [4,5]. 

**Scheme 1:**
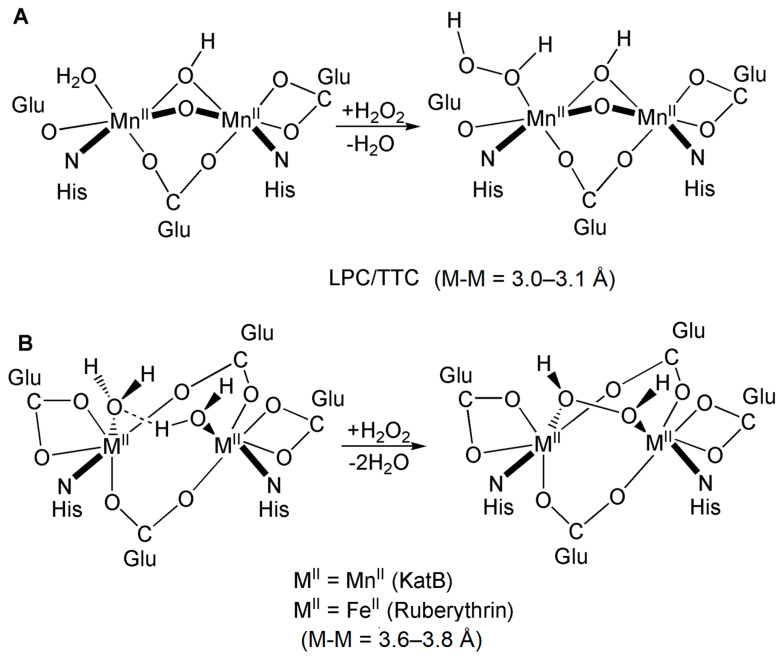
Geometric structures of dinuclear manganese and iron catalases (LPC/TTC (**A**) and *KatB*/Ruberythrin (**B**)), including their native and H_2_O_2_ complexes.

The involvement of manganese in oxygen metabolism is well known, e.g., in manganese superoxide dismutase with a mononuclear manganese and the photosynthetic oxygen-evolving complex with a tetranuclear manganese complexes in their active sites. Manganese catalases are generally rare compared with their type I heme-containing counterparts but are widely found in prokaryotic life. The manganese-containing nonheme type II catalases can be found in various microorganisms including the lactic acid bacteria (e.g., *Lactobacillus plantarum* (LPC) [19,20]) and *thermophiles* (e.g., *Thermus thermophilus* and *Thermoleophilum album* [21,22,23,24,25]). These enzymes differ in their active site, which leads to different biological functions; the *L. plantarum* enzyme can act as catalase, whereas the *T. thermophilus* (TTC) enzyme can function as a catalase/peroxidase (Scheme 1A). These enzymes (so-called “pseudocatalases”) contain a dinuclear manganese active site that changes between the reduced Mn_2_(II,II) and oxidized Mn_3_(III,III) forms during the catalytic cycles, which can be interpreted through the formation of a terminal peroxo intermediate and its activation via O–O bond cleavage [20,25]. Since the heme-containing catalase forms an extremely reactive intermediate that can oxidize protein side chains in the absence of hydrogen peroxide, manganese catalases whose active site is stable both in oxidized and reduced forms may provide an advantage at low hydrogen peroxide levels [26].

In contrast to their structure, the cyanobacterial manganese catalase (*KatB*) from Anabaena shows a significantly different active site that shows high similarity to the active site of the ferritin-like ruberythrin (*RBR*) enzymes [27]. *RBR*s are nonheme diiron proteins, found in many organisms and serve as peroxide scavengers in defense against oxidative stress similarly to Mn catalases. The Mn–Mn distance in *KatB* (~3.8 Å) is almost identical to that observed for iron-containing ribonucleotide reductase *R2* (3.6–3.7 Å). In contrast to the TTC and LPC enzymes with short Mn–Mn distances (3.0–3.1 Å), the symmetric active sites and the relatively large M–M distances in *KatB* and *R2* favor the *μ*-1,2-binding mode of the peroxide (Scheme 1B). The high degree of similarity of the active centers of these enzymes presupposes similar reactive intermediates and similar reaction mechanisms. The relatively large M–M distance in the enzyme *KatB* and *R2* may lead to the homolysis of the peroxo intermediate, resulting in the formation of reactive oxoiron(IV)/oxomanganese(IV) species. Based on this hypothesis, a mechanism similar to oxoiron(IV)-containing heme catalases cannot be ruled out. 

Harmful effects of H_2_O_2_ on biological processes such as aging, cancer and neurodegenerative diseases have been confirmed by numerous studies. These findings led to the development of synthetic catalase mimetics as therapeutic agents that may be effective against oxidative stress in human diseases such as cancer, Alzheimer’s disease, aging and inflammatory and heart diseases. Among them, manganese-based mimics are the most widely investigated, mainly because of their low toxicity (Mn is not prone to generate HO• radical in Fenton type reactions); however, there are several iron- and copper-based systems too. A number of mechanistic and spectroscopic studies have been reported on the catalase activity of non-porphyrin mono- and dinuclear complexes with various ligands including Schiff bases, corroles, polyamines and polypyridyl derivatives. These papers mainly focused on the comparison of mono and dinuclear copper, manganese and iron complexes and the effect of various factors such as ligand donor sites, endogenous acid/base groups and steric parameters [28,29,30,31,32,33,34,35,36,37].

**Scheme 2:**
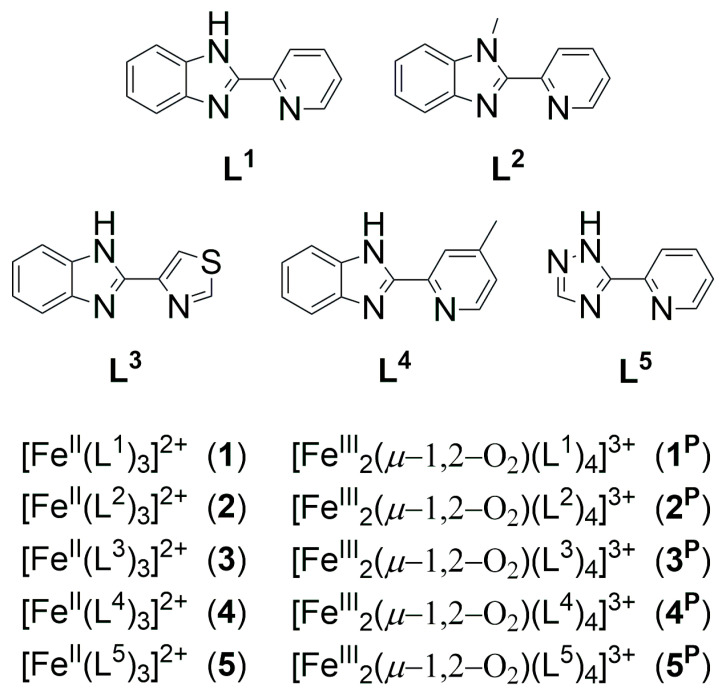
Structure of the ligands and their complexes involved in this study.

As a possible structural and functional model of dimanganese and diiron (*RBR*) catalase enzymes, we have recently investigated the reactivity and the mechanism of two different kinds of peroxo-diiron(III) adducts, namely [Fe^III^_2_(*μ*-O_2_)(L^1−2^)_4_(CH_3_CN)]^4+^ (L^1^ = 2-(2′-pyridyl)-1*H*-benzimidazole, L^2^ = 2-(2′-pyridyl)-*N*-methyl-benzimidazole) [38,39,40,41] and [Fe^III^_2_(*μ*-O)(*μ*-1,2-O_2_)(IndH)_2_(Solv)_2_]^2+^ (IndH = 1,3-bis(2-pyridyl-imino)-isoindolines), with H_2_O_2_ and compared their mechanisms [42,43,44,45]. In the first case we have obtained direct kinetic and computational evidence for the formation of low-spin oxoiron(IV) via the dissociation process [40], whereas in the second case the peroxo-diiron(III) intermediate was the key intermediate [45]. As a continuation of these studies, five heterobidentate-ligand-containing model compounds, including the previously reported and fully characterized [Fe^II^(L^1−2^)_3_]^2+^ [39], [Fe^II^(L^3^)_3_]^2+^ [46] and [Fe^II^(L^4^)_3_]^2+^ [47] (L^1^ = 2-(2′-pyridyl)-1*H*-benzimidazole, L^2^ = 2-(2′-pyridyl)-*N*-methyl-benzimidazole, L^3^ = 2-(4-thiazolyl)-1*H*-benzimidazole and L^4^ = 2-(4′-methyl-2′-pyridyl)-1*H*-benzimidazole) and the novel [Fe^II^(L^5^)_3_]^2+^ (L^5^ = 2-(1*H*-1,2,4-triazol-3-yl)pyridine), were selected to investigate the mechanism and the effect of the redox potential and the spin-state of the catalyst on the reactivity towards H_2_O_2_ (Scheme 2).

## 2. Results and Discussions

**Figure 1:**
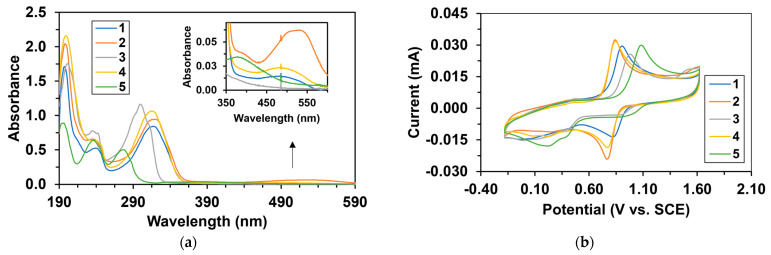
Characterization of [Fe^II^(L^1^-L^5^)]_3_](CF_3_SO_3_)_2_ (**1**–**5**) (L^1^ = 2-(2′-pyridyl)- 1*H*-benzimidazole; L^2^ = 2-(2′-pyridyl)-*N*-methyl-benzimidazole; L^3^ = 2-(4-thiazolyl)-1*H*-benzimidazole; L^4^ = 2-(4′-methyl-2′-pyridyl)-1*H*-benzimidazole; L^5^ = 2-(1*H*-1,2,4-triazol-3-yl)-pyridine) complexes. (**a**) Electronic spectra of [Fe^II^(L^1^-L^5^)]_3_](CF_3_SO_3_)_2_ (**1**–**5**) complexes: [**1**–**5**] = 2.0 × 10^−5^ M, T = 293 K, l = 1 cm, V = 2 mL MeCN. (**b**) Cyclic voltammograms of [Fe^II^(L^1^-L^5^)]_3_](CF_3_SO_3_)_2_ (**1**–**5**) complexes: [**1**–**5**] = 1.0 × 10^−3^ M, in (0.1 M TBAClO_4_) MeCN (10 cm^3^), scan rate: 500 mV/s.

The UV–Vis spectra of complexes **1, 2** and **4** in acetonitrile showed two major features, a band around 380–550 nm and a stronger band around 270–370 nm (Figure 1a). These bands are typical for pyridine-based ligands containing low-spin iron(II) complexes and can be assigned to metal-to-ligand charge transfer (MLCT) and ligand-centered π to π* transitions, respectively [39,48,49,50,51]. In contrast to the low-spin complexes **1**, **2** and **4**, in solution, **3** and **5** show no absorption in the visible region, which is consistent with the latter complexes’ high-spin electronic configuration [46]. A general bathochromic effect was observed as the number of aromatic rings increased in the ligand framework (see L**^1^**, L**^2^** and L**^4^** compared with L**^5^**). This phenomenon can be explained by an important delocalization of π electrons promoted by the linear annelation. Spectroscopic and redox data for complexes **1** to **5** are summarized in Table 1. 

**Figure 2:**
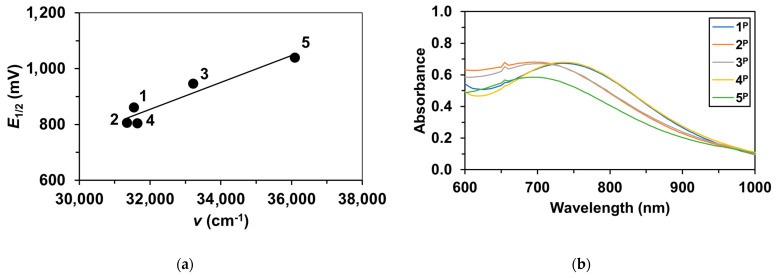
(**a**) Correlation between the redox potential (*E*_1/2_) and the metal-to-ligand charge transfer (MLCT) bands of [Fe^II^(L^1^-L^5^)]_3_](CF_3_SO_3_)_2_ (**1**–**5**) complexes. (**b**) Characteristic UV–Vis spectra of [Fe^III^_2_(*μ*-O_2_)(L^1−5^)_4_(Solv)_2_]^3+^ (**1^P^**–**5^P^**) complexes generated by excess of H_2_O_2_: [**1**–**5**]_0_ = 0.5 × 10^−3^ M, T = 298 K, l = 1 cm, V = 2 mL MeCN.

Complexes **1**–**5** exhibit a quasi-reversible redox couple at ca. 860.5, 805.5, 946, 804 and 1039 mV vs. SCE, respectively (Figure 1b). The Fe^III^/Fe^II^ redox couples for **3** and **5** are at considerably higher potentials than for **2** and **4**. The electrochemical data confirm that in compounds **3** and **5** the iron centers are stronger Lewis acids than those in **2** and **4**, which can be explained with the electron withdrawing nature of the thiazolyl and triazolyl groups compared with pyridyl and benzimidazole, respectively. Introducing an electron-donating methyl substituent at the 5-position of the pyridine rings (**4**) shifted the half-way potential to about 56.5 mV more negative than that of **1**, strengthening the electron density of Fe^III^ d-orbitals and making oxidation less facile. Almost the same effect was observed in the case of the *N*-methylated benzimidazole arm of **2** (55 mV). A linear correlation was found between the energy of the metal-to-ligand charge transfer (MLCT) band and the half-wave potentials, indicating that the substitutions performed on the ligand greatly influenced the electron density of the metal center and thereby its redox properties (Figure 2a). These data can also serve as a basis to investigate possible correlations between the half-wave potentials of the catalysts and their catalytic activities.

**Figure 3:**
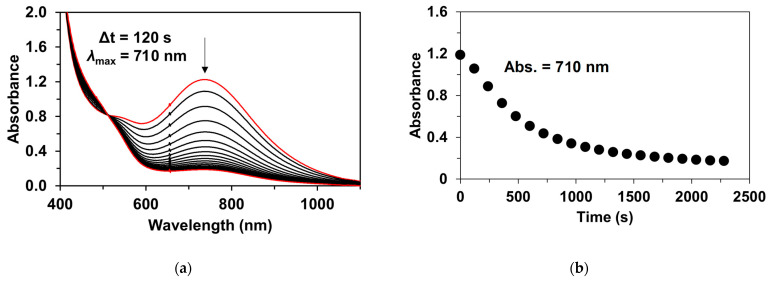
Self-decay of [Fe^III^_2_(*μ*-O_2_)(L^2^)_4_(Solv)_2_]^3+^ (**4^P^**) generated in the reaction of **4** with 4 equivalent of H_2_O_2_. (**a**) UV–Vis spectral change during the decay of **4^P^**. (**b**) Time course of the self-decay of **4^P^**: [**4**]_0_ = 1.0 × 10^−3^ M, [H_2_O_2_]_0_ = 4.0 × 10^−3^ M, l = 1 cm, V = 2 mL MeCN; *k*_sd_ = 1.955 × 10^−3^ s^−1^ for **1^P^** and 1.177 × 10^−3^ s^−1^ for **4^P^** at 298 K.

To date, around 40 peroxo-diiron(III) complexes are known as possible biomimics of diiron enzymes. Similarly to our previously reported [Fe^III^_2_(*μ*-O_2_)(L^1−3^)_4_(CH_3_CN)]^4+^ (**1^P^**-**3^P^**) species, complex **4^P^** and **5^P^** can be generated in acetonitrile with H_2_O_2_. These species, similarly to **1^P^**–**3^P^** (*λ*_max_ = 720 (*ε* = 1360 M^−1^ cm^−1^); *λ*_max_ = 685 (*ε* = 1400 M^−1^ cm^−1^); and *λ*_max_ = 705 (*ε* = 1200 M^−1^ cm^−1^), respectively) [39,46], show characteristic absorption bands at *λ*_max_ = 710 (*ε* = 1360 M^−1^ cm^−1^) and 695 nm (*ε* = 1200 M^−1^ cm^−1^), which can be assigned to the charge transfer between Fe^III^ and the O_2_^2−^ ligand (Figure 2B) [42]. The half-lives (*t*_1/2_) for **1^P^** and **4^P^** generated by four equivalents of H_2_O_2_ at 298 K are 390 s and 500 s, respectively, demonstrating that **4^P^** is more stable than **1^P^**. A similar trend can be observed based on their self-decay data (*k*_sd_ = 1.955 × 10^−3^ s^−1^ for **1^P^** and 1.177 × 10^−3^ s^−1^ for **4^P^**) under identical conditions at 298 K (Figure 3a,b) [40]. The most reactive species is **5^P^**; however, we could not determine its *t*_1/2_ and *k*_sd_ data because its steady-state concentration in the presence of four equivalents of H_2_O_2_ is below 10% at 298 K.

**Figure 4:**
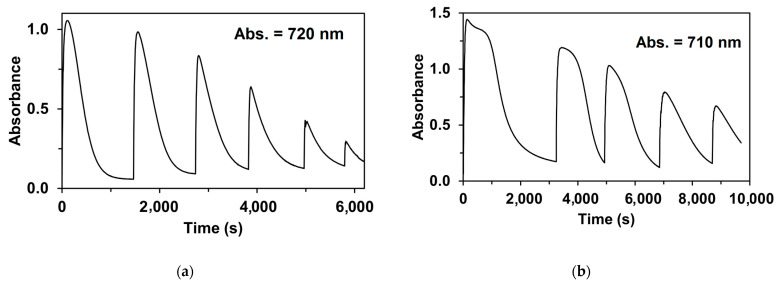
Time course of the formation and decay of *μ*-1,2-peroxo-diiron(III) (**1^P^** and **4^P^**) complexes generated in the reaction of **1** and **4** with 4 equivalent of H_2_O_2_ for 5–6 cycles. (**a**) Generation and decay of **1^P^** for 6 cycles. (**b**) Generation and decay of **4^P^** for 5 cycles. [**1** and **4**]_0_ = 1.0 × 10^−3^ M, [H_2_O_2_] = 4.0 × 10^−3^ M (5-6×), T = 298 K, l = 1 cm, V = 2 mL MeCN.

**Figure 5:**
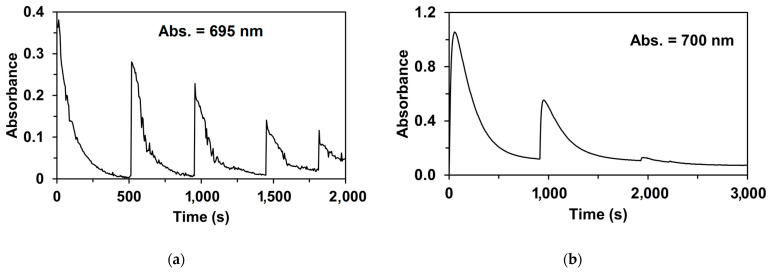
Time course of the formation and decay of *μ*-1,2-peroxo-diiron(III) (**3^P^** and **5^P^**) complexes. (**a**) Time course of the formation and decay of *μ*-1,2-peroxo-diiron(III) (**5^P^**) complex generated in the reaction of **5** with 50 equivalents of H_2_O_2_ for 5 cycles: [**5**]_0_ = 1.0 × 10^−3^ M, H_2_O_2_ = 5.0 × 10^−2^ M, T = 298 K, l = 1 cm, V = 2 mL MeCN. (**b**) Generation and decay of **3^P^** for 3 cycles. [**3**]_0_ = 1.0 × 10^−3^ M, [H_2_O_2_] = 4.0 × 10^−3^ M (3×), T = 298 K, l = 1 cm, V = 2 mL MeCN.

It was also established that by adding additional H_2_O_2_ (four equivalents), **1^P^** and **4^P^** can be regenerated 2–3 times after decomposition (Figure 4a,b) with a relatively high yield (>50%). A similar result was observed in the case of **5^P^** but with much higher H_2_O_2_ (Figure 5a) and a much lower steady-state concentration of **5^P^**. Many fewer cycles and yields can be achieved with **3^P^** (Figure 5b).

**Figure 6:**
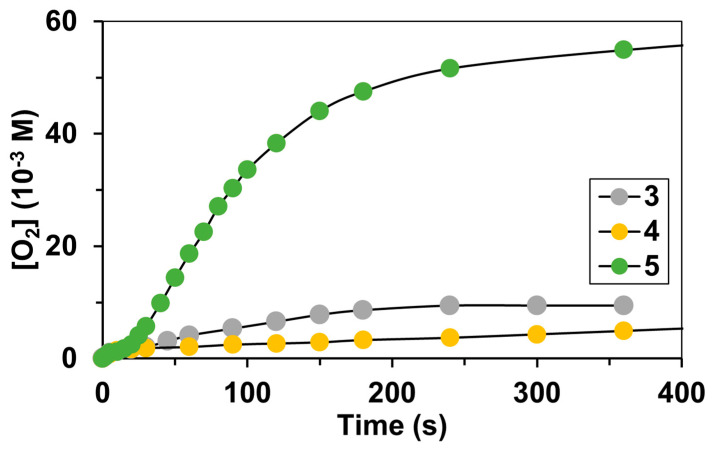
Time traces for the **3^P^**–**5^P^**-mediated disproportionation reaction of H_2_O_2_: [**3**–**5**]_0_ = 1.0 × 10^−3^ M, [H_2_O_2_]_0_ = 300 × 10^−3^ M, T = 298 K, V = 20 mL MeCN.

**Figure 7:**
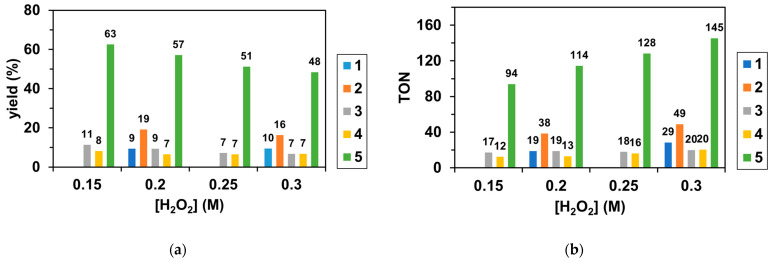
Comparison of the catalase-like activity of [Fe^II^(L^1^-L^5^)]_3_](CF_3_SO_3_)_2_ (**1**–**5**) complexes at 298 K. (**a**) Dependence of the reaction yield on the H_2_O_2_ concentration. (**b**) Dependence of the TON (TON = [H_2_O_2_]/[**1**–**5**] on the H_2_O_2_ concentration (Table 2).

To compare the reactivity of catalysts **1**–**5** with H_2_O_2_, the reactions were carried out under the same conditions ([**1**–**5**]: [H_2_O_2_] = 1: 300 at 298 K), and the amount of dioxygen evolved during the disproportionation of H_2_O_2_ into H_2_O and O_2_ was monitored using a gas volumetric method (Figure 6). Under this condition, the highest catalytic activity can be observed by the use of complex **5**, based on the calculated yield (of dioxygen), TON (TON = turnover number = mol H_2_O_2_/mol catalyst) and TOF (turnover frequency = mol H_2_O_2_/mol catalyst/h calculated as 3600 × (*V*_in_/2/[**1**–**5**]_0_) values (Table 2 and Figure 7). These data for **5** are Yield = 48%, TON = 145 and TOF = 1065 h^−1^. The TOF values for **5** are significantly higher than those observed for previously reported Fe^II^(IndH) systems, including [Fe^III^_2_(*μ*-O)(*μ*-1,2-O_2_)(IndH)_2_(Solv)_2_]^2+^ intermediate formation (~300 h^−1^) [45]. In contrast, the lowest activity was observed in the case of complex **4** with Yield = 6.8%, TON = 20.4 and TOF = 27 h^−1^ under identical conditions. Much more favorable values were observed in all cases at lower substrate (H_2_O_2_) concentrations, which can be explained by the water content (Figure 7a,b). As we noticed before (Figure 4 and Figure 5), the amount of the peroxo complexes decreases significantly with the increase in water, which suggests that the **1^P^**–**5^P^** intermediates play a key role in the catalytic cycles.

**Figure 8:**
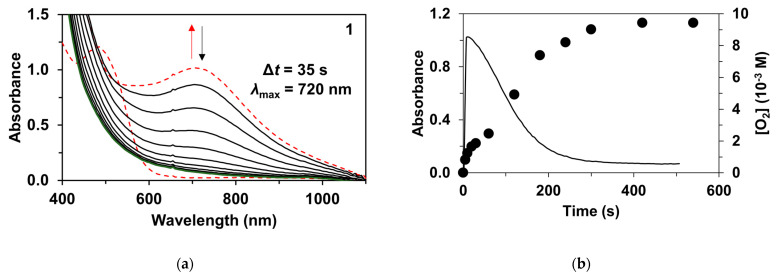
(**a**) UV–Vis spectral changes of **1^P^** during the catalytic disproportionation reaction of H_2_O_2_. (**b**) Time course of the formation and decay of **1^P^** during the catalytic disproportionation reaction of H_2_O_2_ and the parallel formation of dioxygen monitored by UV–Vis spectroscopy at 720 nm and by volumetrically determined amounts of evolved dioxygen. [**1**]_0_ = 1.0 × 10^−3^ M, [H_2_O_2_]_0_ = 0.3 M, T = 298 K in MeCN.

**Figure 9:**
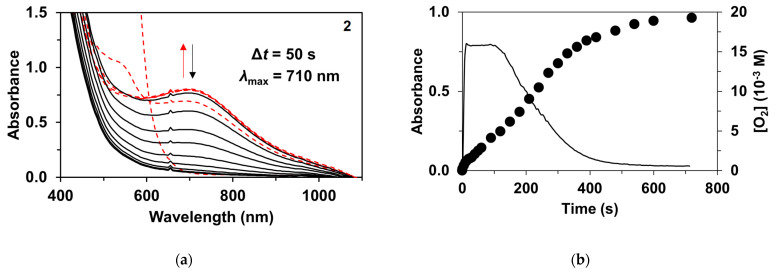
(**a**) UV–Vis spectral changes of **2^P^** during the catalytic disproportionation reaction of H_2_O_2_. (**b**) Time course of the formation and decay of **2^P^** during the catalytic disproportionation reaction of H_2_O_2_ and the parallel formation of dioxygen monitored by UV–Vis spectroscopy at 710 nm and by volumetrically determined amounts of evolved dioxygen. [**2**]_0_ = 1.0 × 10^−3^ M, [H_2_O_2_]_0_ = 0.3 M, T = 298 K in MeCN.

**Figure 10:**
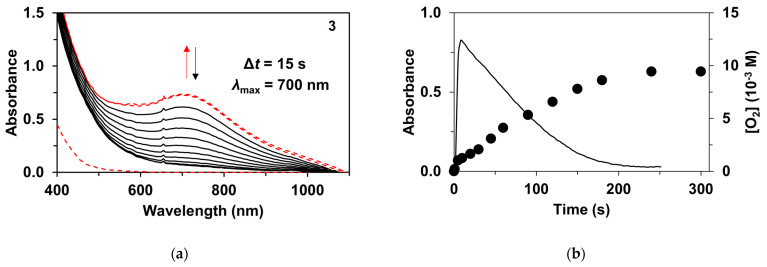
(**a**) UV–Vis spectral changes of **3^P^** during the catalytic disproportionation reaction of H_2_O_2_. (**b**) Time course of the formation and decay of **3^P^** during the catalytic disproportionation reaction of H_2_O_2_ and the parallel formation of dioxygen monitored by UV–Vis spectroscopy at 700 nm and by volumetrically determined amounts of evolved dioxygen. [**3**]_0_ = 1.0 × 10^−3^ M, [H_2_O_2_]_0_ = 0.3 M, T = 298 K in MeCN.

**Figure 11:**
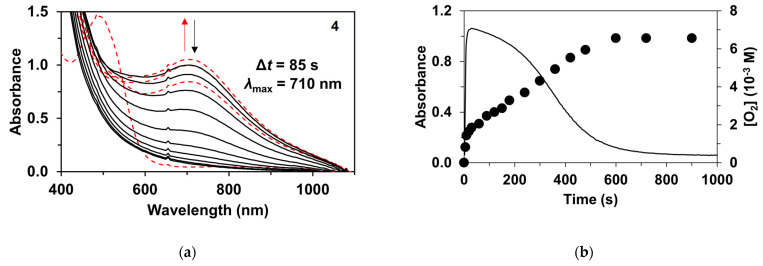
(**a**) UV–Vis spectral changes of **4^P^** during the catalytic disproportionation reaction of H_2_O_2_. (**b**) Time course of the formation and decay of **4^P^** during the catalytic disproportionation reaction of H_2_O_2_ and the parallel formation of dioxygen monitored by UV–Vis spectroscopy at 710 nm and by volumetrically determined amounts of evolved dioxygen. [**4**]_0_ = 1.0 × 10^−3^ M, [H_2_O_2_]_0_ = 0.3 M, T = 298 K in MeCN.

**Figure 12:**
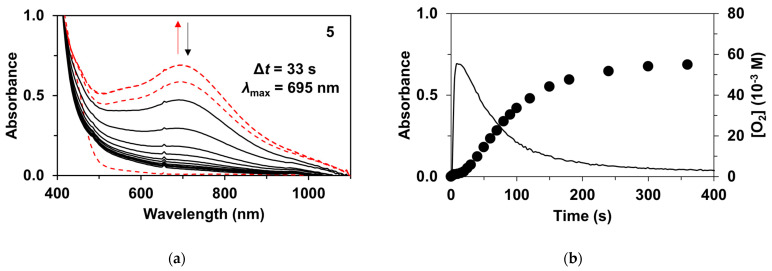
(**a**) UV–Vis spectral changes of **5^P^** during the catalytic disproportionation reaction of H_2_O_2_. (**b**) Time course of the formation and decay of **5^P^** during the catalytic disproportionation reaction of H_2_O_2_ and the parallel formation of dioxygen monitored by UV–Vis spectroscopy at 695 nm and by volumetrically determined amounts of evolved dioxygen. [**5**]_0_ = 1.0 × 10^−3^ M, [H_2_O_2_]_0_ = 0.3 M, T = 298 K in MeCN.

To prove this assumption, we followed the formation and decomposition of peroxo intermediates (rise and fall of their visible chromophores at *λ*_max_ = 720 (Figure 8a), 710 (Figure 9a), 700 (Figure 10a), 710 (Figure 11a) and 695 nm (Figure 12a)), as well as the appearance of evolving dioxygen during the disproportionation reaction of H_2_O_2_, using parallel UV–Vis and gas volume measurement methods (Figure 8b, Figure 9b, Figure 10b, Figure 11b and Figure 12b). Based on the above measurements, the profile of the catalytic reactions can be divided into two main steps, namely a short lag phase with the formation and accumulation of peroxo intermediates, followed by their decomposition with linear dioxygen formation.

Detailed kinetic studies of the in situ formed *μ*-1,2-peroxo-diiron(III)-mediated disproportionation reaction of H_2_O_2_ were carried in MeCN at 298 K under pseudo-first-order conditions with an excess of H_2_O_2_ ([**3**–**5**]_0_: [H_2_O_2_]_0_ = 0.5–2.5 mM: 150–300 mM) using an initial rate method monitoring the increase in the evolved dioxygen by gas volumetric measurement. The estimated initial rates (under same conditions) according to the reaction rate order are *V*_i_ = 1.51 × 10^−5^ M s^−1^, *V*_i_ = 1.86 × 10^−5^ M s^−1^, *V*_i_ = 3.40 × 10^−5^ M s^−1^, *V*_i_ = 5.65 × 10^−5^ M s^−1^ and *V*_i_ = 59.2 × 10^−5^ M s^−1^, respectively to compounds **4**, **1**, **2**, **3** and **5**, respectively, at 298 K. Based on the observed initial rates, compound **5** has significantly higher reactivity than **4**. 

**Figure 13:**
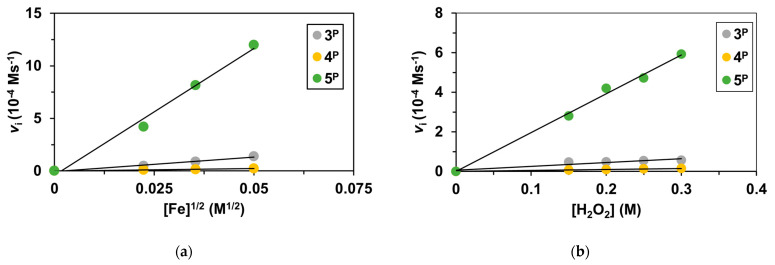
Comparison of the **3^P^**–**5^P^**-mediated disproportionation reaction of H_2_O_2_ in MeCN at 298 K. (**a**) Dependence of the reaction rate (*V*_i_) on the in situ generated [**3^P^**–**5^P^**] concentration: [H_2_O_2_]_0_ = 0.3 M. (**b**) Dependence of the reaction rate (*V*_i_) on the [H_2_O_2_]_0_ concentration: [**3^P^**–**5^P^**] = 0.5 mM.

In order to determine the rate dependence on the various reactants, disproportionation runs were performed at different substrate and catalyst concentrations (Table 3). At constant [H_2_O_2_]_0_, the initial rate of H_2_O_2_ disproportionation varies linearly with the in situ-formed [catalyst **3^P^, 4^P^** or **5^P^**]^0.5^, meaning that all reactions are half-order in catalyst and suggesting a dissociation process via homolytic cleavage of the O─O bond (Figure 13a), similarly to the previously published **1** and **2** H_2_O_2_ systems [40]. However, at low H_2_O_2_ concentrations, the reactions are first-order in peroxide concentration as shown in Figure 13b, to establish a rate law of +d[O_2_]/d*t* = *k*_cat_[H_2_O_2_][**1^P^**-**5^P^**]^0.5^ with *k*_cat_ = 2.81 × 10^−3^ M^−1/2^ s^−1^ for **1^P^** [40], 5.06 × 10^−3^ M^−1/2^ s^−1^ for **2^P^** [40], *k*_cat_ = 11.3 × 10^−3^ M^−1/2^ s^−1^ for **3^P^**, *k*_cat_ = 2.16 × 10^−3^ M^−1/2^ s^−1^ for **4^P^** and *k*_cat_ = 96.1 × 10^−3^ M^−1/2^ s^−1^ for **5^P^** at 25 °C.

**Figure 14:**
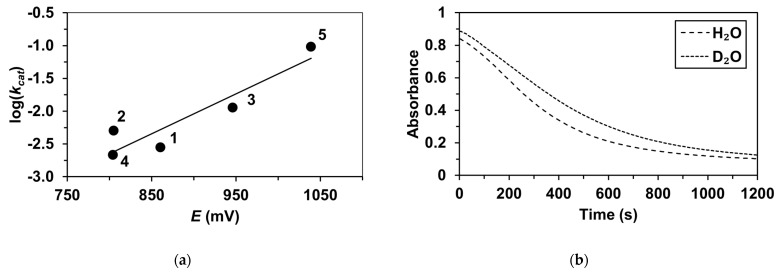
Peroxo-diiron(III)-mediated disproportionation of H_2_O_2_ in MeCN at 298 K. (**a**) Dependence of the reaction rate (*k*_cat_) for H_2_O_2_ oxidation on the redox potential (*E*_1/2_) of the [Fe^II^(L^1−5^)_3_]^2+^ (**1**–**5**) complexes. (**b**) Kinetic isotope effect (*KIE*) on the disproportionation of H_2_O_2_. [**4**]_0_ = 1.0 × 10^−3^ M, [H_2_O_2_]_0_ = 0.05 M, [H_2_O or D_2_O]_0_ = 2.8 M (100 μL) in MeCN.

In the next step, the effect of ligand modification on the reaction rate was investigated by introducing an electron-donating (-CH_3_) substituent into the benzimidazole (L^2^, **2**) and phenyl ring (L^4^, **4**), replacing a pyridyl arm with a thiazolyl ((L^3^, **3**) and a benzimidazole arm with triazole (L^5^, **5**). We found that complexes **3** and **5** containing the thiazolyl and triazole side chains are the most effective oxidants with the fastest rates: *k*_cat_ = 11.3 × 10*^−^*^3^ M*^−^*^1/2^ s*^−^*^1^ and 96.1 × 10*^−^*^3^ M*^−^*^1/2^ s*^−^*^1^, respectively. On the other hand, complexes **1** and **4,** with unsubstituted and 4-methylpyridine arms, are the less efficient oxidants with *k_cat_* = 2.81 × 10*^−^*^3^ M*^−^*^1/2^ s*^−^*^1^ and 2.16 × 10*^−^*^3^ M*^−^*^1/2^ s*^−^*^1^, respectively. These results provide clear evidence that the ligand environment of the metal center influences the redox potential and the catalase activity of the complexes. Table 1 and Figure 14a show that the redox potential (E_1/2_) of the complexes and their activity increase with the increase in the electron-withdrawing capacity of the substituent/side-chain. A kinetic isotope effect (*KIE*) of 1.36 was observed for the decay of **4^P^** when the experiments were carried out in the presence of added H_2_O (*k* = 3.68 × 10*^−^*^3^ s*^−^*^1^) or D_2_O (*k* = 2.70 × 10*^−^*^3^ s*^−^*^1^) (Figure 14b). These results indicate the role of the water during the diiron-peroxo-mediated disproportionation reaction.

## 3. Experimental

### 3.1. Materials and Methods

All chemicals including L^1^ = 2-(2-pyridyl)-1*H*-benzimidazole, L^2^ = 2-(2′-pyridyl)-*N*-methyl-benzimidazole, L^3^ = 2-(4-thiazolyl)-1*H*-benzimidazole, L^4^ = 2-(4-Methyl-2-pyridyl)-1*H*-benzimidazole and L^5^ = 2-(1*H*-1,2,4-triazol-3-yl)pyridine ligands were obtained from Aldrich Chemical Co. and used without further purification unless otherwise indicated. Solvents were dried according to the published procedures, distilled and stored under argon. [Fe^II^(L^2^)_3_](CF_3_SO_3_)_2_ (**4**) and [Fe^II^(L^1−3^)_3_](CF_3_SO_3_)_2_ (**1**) were synthesized according to the literature methods [39,46]. UV–visible spectra were recorded on an Agilent 8453 diode-array spectrophotometer using quartz cells. The infrared spectroscopy measurements were carried out using an IRAffinity-1s spectrophotometer with a MIRacle10 (Diamond/ZnSe) one reflection ATR plate and LabSolutions IR Series Software. Cyclic voltammetric experiments were carried out using an SP-150 potentiostat and EC-Lab V11.41 software. During the measurements, we used a three-electrode setup where a 3.0 mm diameter glassy-carbon electrode was the working electrode, a Pt wire was the counter electrode and an Ag/Ag^+^ (1M TBAClO_4_ in acetonitrile) was the reference electrode. The supporting electrolyte was a 0.1 M solution of tetrabutylammonium perchlorate.

### 3.2. Synthesis of [Fe^II^(L^3^)_3_](CF_3_SO_3_)_2_ (5)

The Fe^II^(CF_3_SO_3_)_2_ (0.67 g, 1.9 mmol) was dissolved in dry acetonitrile (15 mL) under an Ar atmosphere. Then, the 2-(1*H*-1,2,4-triazol-3-yl)-pyridine, L^5^ (0.823 g, 5.7 mmol) was added to the stirred solution. The color of the solution turned brown. The solution was stirred for 4 h under an Ar atmosphere. The solution was layered with diethyl ether (15 mL) after the complete diffusion the solvent was removed and the precipitated yellow solid was washed again with diethyl ether and dried in vacuum. Yield: 1.41 g (94%). Anal. Calcd. for C_21_H_18_F_6_FeN_12_O_6_S_2_: C, 51.03; H, 3.67; N, 34.00. Found: C, 51.23; H, 3.54; N, 34.13. FT-IR bands (ATR, cm^−1^): 3145 w, 2926 w, 1610 w, 1504 w, 1481 w, 1456 w, 1431 w, 1394 w, 1350 w, 1309 m, 1288 s, 1232 s, 1209 s, 1159 s, 1087 w, 1053 w, 1028 s, 999 m, 975 m, 854 w, 798 m, 746 m, 725 s, 632 s. UV–Vis absorption (CH_3_CN) [*λ*_max_ nm, (log*ε*)] 196 (4.65), 236 (4.51), 277 (4.4), 372 (3.27).

### 3.3. Gas Volumetric Measurements

The catalase-like activity was investigated through the evolution of dioxygen. The reactions were carried out in a 30 cm^3^ reactor under air at 25 °C. In a typical experiment, 4 × 10^−5^ mol of [Fe^II^(L^3^)_3_](CF_3_SO_3_)_2_, [Fe^II^(L^4^)_3_](CF_3_SO_3_)_2_ and [Fe^II^(L^5^)_3_](CF_3_SO_3_)_2_ (**3**/**4**/**5**) was dissolved in 20 cm^3^ CH_3_CN and the reactor was closed with a rubber septum. The flask was connected to a graduated burette filled with oil. The right amount of H_2_O_2_ was added to the reactor through the septum and the reaction was followed by the amount of the dioxygen evolved. At constant [H_2_O_2_]_0_, the reactions exhibit ½-order kinetics on catalysts (in situ formed **1^P^**–**5^P^**) and the ½-order rate constants (*k*_1′_) were obtained from the slope of the plot of *V*_i_ (after the leg phase) vs. [**1^P^**–**5^P^**]. At fixed [**1^P^**–**5^P^**], reaction rates showed a good linear dependence with [H_2_O_2_]_0_, affording first-order rate constants (*k*_1′_’). The catalytic constants were obtained from either *k*_1′_/[H_2_O_2_]_0_ or *k*_1′_’/[**1^P^**–**5^P^**]^1/2^.

### 3.4. UV–Vis Spectroscopic Measurements

The reactive species [Fe^III^_2_(*μ*-1,2-O_2_)(L^1−5^)_4_] (**1^P^**–**5^P^**) were generated by adding 150–300 equivalent H_2_O_2_ to the solutions of (**1**–**5**) in a 1 cm quartz UV cuvette at 25 °C. The total volume was 2 cm^3^ (in CH_3_CN). The formation and the decay of (**1^P^**–**5^P^**) was monitored with a UV–Vis spectrophotometer at 720, 710, 700, 710 and 695 nm, respectively. In a typical experiment, 4 × 10^−5^ mol of (**1**–**5**) was dissolved in 2 cm^3^ CH_3_CN and the right amount of H_2_O_2_ was added to the cuvette. Reactions were run at least in triplicate, and the data reported are the average of the reactions.

## 4. Conclusions

**Scheme 3:**
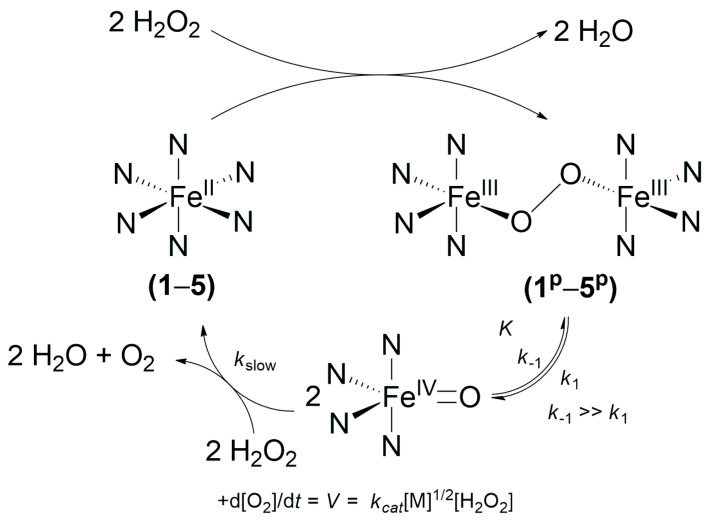
Schematic mechanism of the iron-mediated disproportionation reaction of H_2_O_2_.

The reactivity of three iron(II) complexes (**3**, **4** and **5**) with heterobidentate ligands compared with the previously reported **1** and **2** have been investigated in the disproportionation reaction of H_2_O_2_ as possible biomimics of catalase enzymes. It can be concluded that the formation of the corresponding *μ*-1,2-peroxo-diiron(III) complexes (**1^P^**–**5^P^**) and their catalase-like activity can be clearly verified in the case of all three precursor complexes (**1**–**5**). Similar to our previous kinetic results on the **1^P^**(**2^P^**)-containing system, we have got a half-order for **3^P^**, **4^P^** and **5^P^**, which further confirms the dissociative mechanism, including the homolytic O–O cleavage and the formation of mononuclear oxoiron(IV) species. The formation of oxoiron(IV) can be described by a pre-equilibrium process, which is shifted in the direction of the starting peroxo complex. Its presence in a steady-state concentration is also supported by previous theoretical calculations [40]. During fine-tuning of the catalyst, we found clear evidence that the electron-deficient sites (**3** and **5**) are significantly more reactive, the higher the redox potentials of the Fe^III^/Fe^II^ redox couple, the higher the catalase-like activity. Furthermore, it is important to note that the two most active catalysts with L^3^ and L^5^ ligands have a high-spin electronic configuration. This is in good agreement with the electrophilic nature of the proposed oxoiron(IV) species during the catalytic cycle. Based on the obtained results, the proposed route may represent a new alternative to the mechanism of dinuclear catalase systems (Scheme 3). 

## Figures and Tables

**Table 1:** Summary of spectroscopic and redox potential data of iron(II) precursor complexes compared with their catalase-like activity.

Catalyst	*λ*_1_^Fe(II)^ (nm)	ν_1_^Fe(II)^ (cm^−1^)	*E*_pa_ (mV)	*E*_pc_ (mV)	*E*_1/2_ (mV)	*k*_cat_^1^ (10^−3^M^−1/2^s^−1^)
**1**	317	31,546	898	823	860.5	2.81 ^2^
**2**	319	31,348	840	771	805.5	5.06 ^2^
**3**	301 ^3^	33,223	970	922	946	11.3
**4**	316	31,646	840	768	804	2.16
**5**	277	36,101	1083	996	1039	96.6

^1^ Measurements carried out in MeCN at 298 K (see kinetic part). ^2^ [40]. ^3^ [46].

**Table 2:** Comparison of the catalase-like activity of [Fe^II^(L^1^-L^5^)]_3_](CF_3_SO_3_)_2_ (**1**–**5**) complexes at 298 K.

Catalyst	Yield (%)	TON/Fe [H_2_O_2_]_t_/[Catalyst]_0_	TOF * [H_2_O_2_]_t_/[Catalyst]_0_/h
**1**	9.5	28.5	53
**2**	16.3	48.9	61
**3**	6.7	20.0	102
**4**	6.8	20.4	27
**5**	48.4	145	1065

* Calculated from the *V*_i_ values: TOF = 3600 × (*V*_i_/2/[**1**-**5**]): [**1**-**5**]_0_ = 1.0 × 10^−3^ M, [H_2_O_2_]_0_ = 300 × 10^−3^ M, T = 298 K.

**Table 3:** Kinetic data for the diiron(III)-mediated (**3^P^**–**5^P^**) disproportionation reaction of H_2_O_2_ derived from gas volumetric measurements in MeCN at 298 K.

Catalyst	[Catalyst]_0_ (10^−3^M)	[H_2_O_2_]_0_ (M)	*V*_i_^1^ (10^−5^Ms^−1^)	*k_cat_*^2^ (10^−3^M^−1/2^s^−1^)
**3^P^**	0.50	0.20	4.78	10.7
	1.25	0.20	8.63	12.2
	2.50	0.20	13.8	13.8
	0.50	0.15	4.64	13.8
	0.50	0.20	4.78	10.7
	0.50	0.25	5.35	9.57
	0.50	0.30	5.65	8.42
				**11.3**
**4^P^**	0.50	0.20	0.95	2.14
	1.25	0.20	1.50	2.12
	2.50	0.20	2.39	2.39
	0.50	0.15	0.65	1.94
	0.50	0.20	0.95	2.14
	0.50	0.25	1.21	2.16
	0.50	0.30	1.51	2.25
				**2.16**
**5^P^**	0.50	0.20	40.4	90.3
	1.25	0.20	81.5	115
	2.50	0.20	120	120
	0.50	0.15	28.1	83.8
	0.50	0.20	40.4	90.3
	0.50	0.25	47.2	84.4
	0.50	0.30	59.2	88.3
				**96.1**

^1^ +d[O_2_]/d*t* = *V* = *k*_cat_ [**3^P^**–**5^P^**]^1/2^[H_2_O_2_]. ^2^ *k*_cat_ = V/([H_2_O_2_] × [**3^P^**–**5^P^**]).

## Data Availability

Not available.

## Sample Availability

Samples of the compounds are not available.
